# Supplementary material for: Effects of Long-Term Protein Restriction on Meat Quality and Muscle Metabolites of Shaziling Pigs
Source: Animals (Basel). 2022 Aug 8;12(15):2007. doi: 10.3390/ani12152007 (PMC9367386; doi:10.3390/ani12152007)
Supplement: Supplementary file 1 [file animals-12-02007-s001.zip › PDF S1.pdf]

ICS 65.020.30
B 43

NY

中华人民共和国农业行业标准

NY/T 2826—2015

沙 子 岭 猪

Shaziling pig

2015-10-09 发布　　　　2015-12-01 实施

中华人民共和国农业部 发布

前　言

本标准按照 GB/T 1.1—2009 给出的规则起草。

本标准由农业部畜牧业司提出。

本标准由全国畜牧业标准化技术委员会(SAC/TC 274)归口。

本标准起草单位:湘潭市畜牧兽医水产局、全国畜牧总站、湖南农业大学、湘潭市家畜育种站、湘潭飞龙牧业有限公司。

本标准主要起草人:吴买生、刘彬、张彬、左晓红、薛立群、向拥军、刘伟、刘传芳、李朝晖、张善文。

沙　子　岭　猪

1　范围

本标准规定了沙子岭猪的品种特征特性、生产性能测定及种猪出场要求等内容。

本标准适用于沙子岭猪品种鉴别。

2　规范性引用文件

下列文件对于本文件的应用是必不可少的。凡是注日期的引用文件，仅注日期的版本适用于本文件。凡是不注日期的引用文件，其最新版本(包括所有的修改单)适用于本文件。

GB 16567　种畜禽调运检疫技术规范

NY/T 820　种猪登记技术规范

NY/T 821　猪肌肉品质测定技术规范

NY/T 822　种猪生产性能测定规程

3　产地与分布

沙子岭猪是华中两头乌猪的一个类群，原产于湖南省湘潭市。中心产区在湘潭县的云湖桥、花石、青山桥、石鼓，湘乡市的月山、白田、龙洞，韶山市的大坪、杨林、如意，雨湖区的姜畬、响塘以及衡阳县的洪市、大安、曲兰和常宁市的荫田等 20 多个乡(镇)。主要分布于湖南湘潭、衡阳、永州、娄底、邵阳等市的 10 多个县(市、区)。

4　体型外貌

4.1　外貌特征

体型有大型和小型之分，现存沙子岭猪以大型为主。被毛较粗稀，毛色为“点头墨尾”，即头部毛和臀部毛为黑色，黑白交界处有“晕”，其他部位为白色，部分猪背腰部有隐花。头短而宽，嘴筒齐，面微凹，耳中等大、蝶形，额部有皱纹。背腰较平直，腹大不拖地。腿臀欠丰满，四肢粗壮结实，后肢开张。乳头数 6 对～8 对。沙子岭猪体型外貌参见附录 A。

4.2　体重体尺

参照附录 B 的营养水平饲养，6 月龄公猪平均体重 42 kg，体长 90 cm，体高 45 cm；6 月龄母猪平均体重 45 kg，体长 87 cm，体高 42 cm。成年公猪平均体重 130 kg，体长 136 cm，体高 71 cm；成年母猪平均体重 145 kg，体长 138 cm，体高 66 cm。

5　繁殖性能

公猪性成熟期为 3 月龄，初配年龄为 5 月龄～6 月龄。母猪初情期在 3.5 月龄，适配期为 5 月龄～6 月龄。初产母猪平均总产仔数 9 头，产活仔数 8.5 头，平均初生个体重 0.8 kg，21 日龄窝重 24 kg，35 日龄断奶窝重 32 kg；经产母猪平均总产仔数 11.5 头，产活仔数 10.6 头，平均 21 日龄窝重 28 kg，35 日龄断奶窝重 45 kg。

6　肥育性能

参照附录 B 的营养水平饲养，15 kg～85 kg 生长肥育期间平均日增重 450 g，料重比 4.3∶1。商品猪适宜屠宰体重为 75 kg～85 kg。

7 胴体性能

肥育猪在平均体重 85 kg 屠宰时，平均屠宰率 72%，瘦肉率 41%，眼肌面积 21 cm^2，腿臀比例 25%；平均背膘厚 45 mm。

8 肌肉品质

肥育猪在平均体重 85 kg 屠宰时，肌肉 pH 6.1～6.4；肉色评分(5 分制)3 分～3.5 分；大理石纹评分(5 分制)3 分～3.5 分；滴水损失 1.6%；肌内脂肪含量 3.5%。

9 测定方法

9.1 生长发育、胴体性能测定按照 NY/T 820、NY/T 822 的规定执行。

9.2 繁殖性能测定按照 NY/T 820 的规定执行。

9.3 肌肉品质按照 NY/T 821 的规定执行。

10 种猪合格评定及出场要求

10.1 体型外貌符合本品种特征。

10.2 生殖器官发育正常。有效乳头数不少于 6 对。

10.3 无遗传疾患和损征。

10.4 来源、血缘和个体标识清楚，系谱记录齐全。

10.5 按照 GB 16567 的要求检疫合格。

附　录　A
（资料性附录）
沙子岭猪照片

A.1　沙子岭猪头部

见图 A.1。

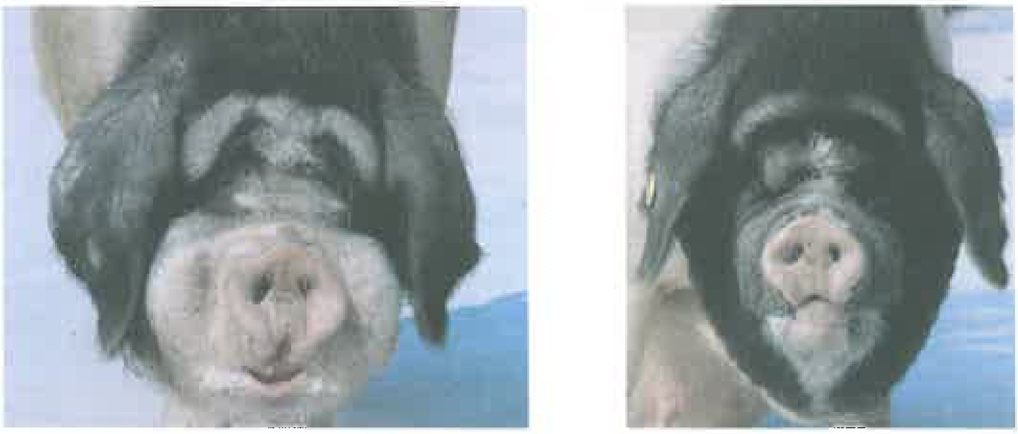

公猪　　母猪

图 A.1　沙子岭猪头部

A.2　沙子岭猪侧部

见图 A.2。

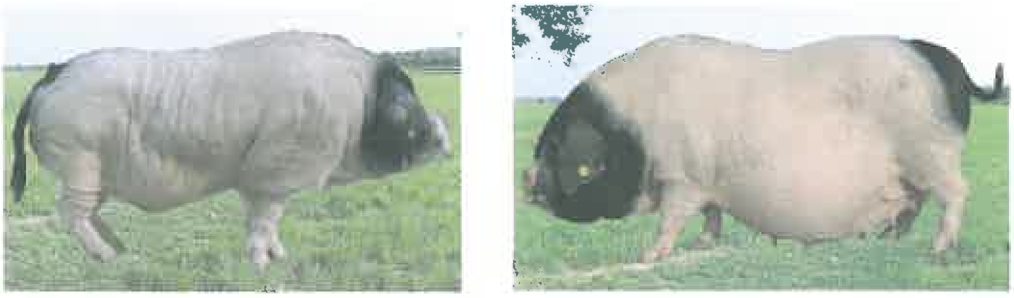

公猪　　母猪

图 A.2　沙子岭猪侧部

A.3　沙子岭猪后部

见图 A.3。

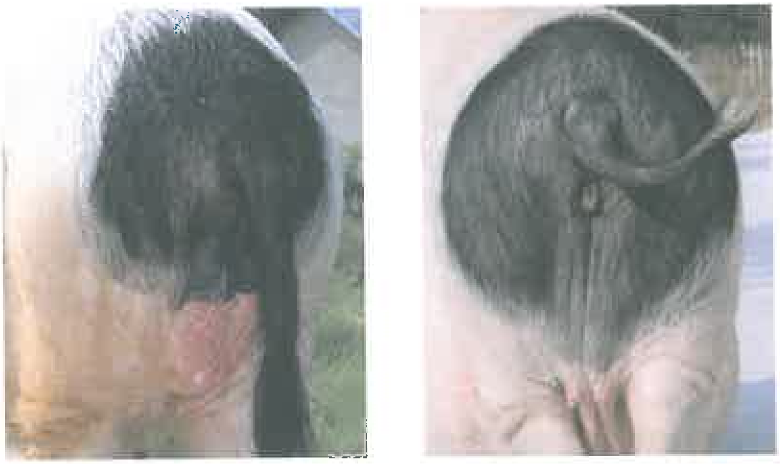

公猪　　　　母猪

图 A.3　沙子岭猪后部

附　录　B
（资料性附录）
沙子岭猪营养需要

沙子岭猪营养需要见表 B.1。

表 B.1　沙子岭猪营养需要

类　　别	阶段<br>kg	消化能<br>MJ/kg	粗蛋白<br>%	钙<br>%	磷<br>%	食盐<br>%
后备种猪	30～50	11.70	13	0.6	0.5	0.3
妊娠母猪	前期	11.29	11	0.61	0.5	0.32
	后期	11.70	13	0.61	0.5	0.32
哺乳母猪		12.54	15	0.64	0.5	0.44
种公猪		12.54	15	0.66	0.5	0.35
仔猪	5～10	13.38	20	0.7	0.6	0.25
	10～15	13.38	18	0.65	0.55	0.25
	15～30	12.54	16	0.55	0.45	0.3
生长育肥猪	30～50	12.12	14	0.55	0.45	0.3
	50 以上	12.70	12	0.5	0.4	0.3
