# Supplementary figures and images for: Effects of Long-Term Protein Restriction on Meat Quality and Muscle Metabolites of Shaziling Pigs
Source: Animals (Basel). 2022 Aug 8;12(15):2007. doi: 10.3390/ani12152007 (PMC9367386; doi:10.3390/ani12152007)

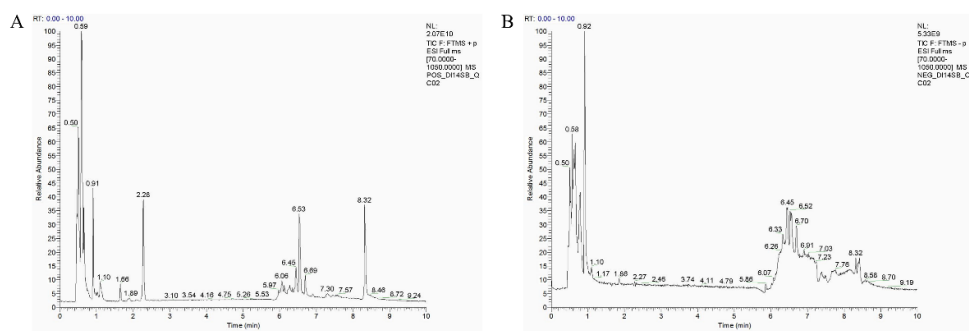

**Figure S1.** The total ion chromatogram of the QC samples in positive (**A**) and negative (**B**) ion modes.

Supplement: Supplementary file 1 [file animals-12-02007-s001.zip › Figure S1.pdf]
